# Intralymphatic histiocytosis in a patient with lung adenocarcinoma treated with pembrolizumab: a case report

**DOI:** 10.1186/s40425-019-0534-z

**Published:** 2019-02-27

**Authors:** Teppei Sugano, Masahiro Seike, Yoko Funasaka, Mai Yoshida, Ryoko Takayama, Ken Okamura, Asuka Nakanishi, Toru Tanaka, Susumu Takeuchi, Rintaro Noro, Yuji Minegishi, Kaoru Kubota, Hidehisa Saeki, Akihiko Gemma

**Affiliations:** 1 Department of Pulmonary Medicine and Oncology, Graduate School of Medicine, Nippon Medical School, 1-1-5, Sendagi, Bunkyo-ku, Tokyo, 113-8603 Japan; 2 Department of Dermatology, Nippon Medical School, Tokyo, Japan

**Keywords:** Lung adenocarcinoma, Pembrolizumab, Intralymphatic histiocytosis

## Abstract

**Background:**

Pembrolizumab, an anti-programmed cell death-1 protein monoclonal antibody, is effective for patients with advanced non-small-cell lung cancer. However, immune checkpoint inhibitors such as pembrolizumab induce various immune-related adverse events, involving the lung, liver, gastrointestinal, endocrine system, and skin. Intralymphatic histiocytosis (ILH) is a rare, chronic cutaneous disorder with a reactive inflammatory component, which often occurs in patients with rheumatoid arthritis.

**Case presentation:**

We present a 67-year-old man with lung adenocarcinoma who developed ILH associated with pembrolizumab treatment. He was treated with palliative thoracic radiotherapy for superior vena cava syndrome. Subsequently, he received four cycles of pembrolizumab. Approximately 2.5 months after the initiation of pembrolizumab, he developed erythema on the trunk of his body. Based on findings of skin biopsies, he was diagnosed with pembrolizumab-induced ILH. Moreover, the upregulation of tumor necrosis factor-α was observed during pembrolizumab therapy.

**Conclusions:**

This is the first report of ILH induced by pembrolizumab in a patient with lung adenocarcinoma.

## Background

Recently, immune checkpoint inhibitors (ICIs) have shown promising results in clinical trials and are recognized as the standard treatment for advanced non-small-cell lung cancer (NSCLC) [1, 2]. Pembrolizumab, an anti-programmed death (PD-1) antibody, has shown favorable antitumor efficacy in NSCLC patients [1, 2]. Of note, patients with high levels of programmed death ligand 1 (PD-L1) expression (tumor proportion score [TPS] ≥ 50%) treated with pembrolizumab had significant survival benefit in untreated metastatic NSCLC [2].

ICIs can induce unique adverse events including pneumonitis, colitis, thyroiditis, and dermatitis, which collectively are termed immune-related adverse events (ir-AEs) [3]. The most frequent cutaneous ir-AEs are maculopapular eruption, lichenoid reactions, pruritus, and vitiligo [4, 5]. Intralymphatic histiocytosis (ILH) is characterized by the presence of dilated lymphatic vessels containing aggregates of mononuclear histiocytes (macrophages) within their lumina in the dermis. It was previously reported that tumor necrosis factor α (TNF-α) is associated with the pathogenesis of ILH. Here, we report the first case of ILH associated with pembrolizumab treatment and the upregulation of TNF-α in a patient with lung adenocarcinoma.

## Case presentation

**Fig. 1:**
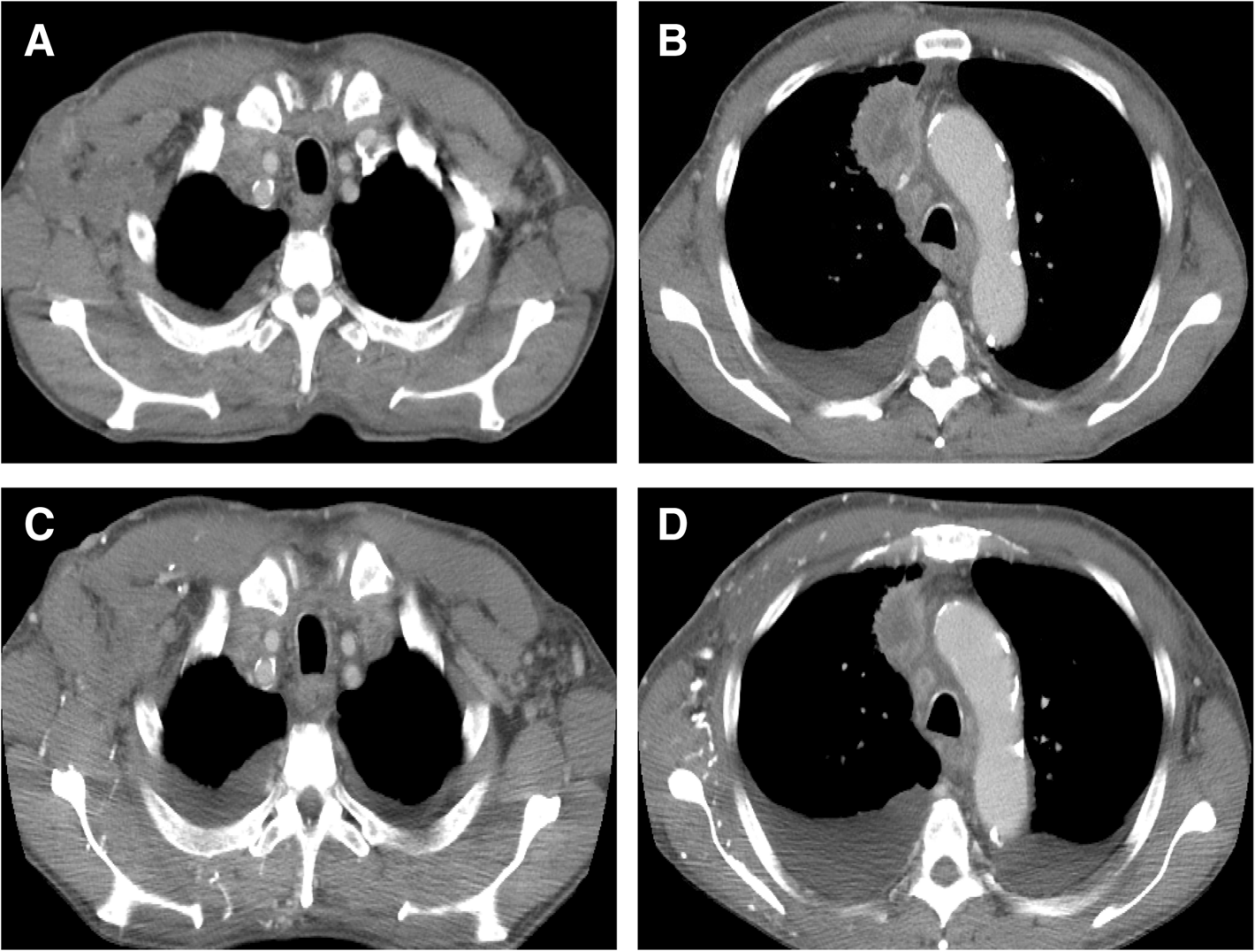
Chest computed tomography analysis determines the baseline before pembrolizumab therapy. A tumor approximately 43 mm in diameter in the right upper lobe, right axial and mediastinal lymph node metastases, and pleural effusion were observed (**a**, **b**). After palliative radiotherapy, the size of the right axial lymph node metastasis was decreased (**c**, **d**)

**Fig. 2:**
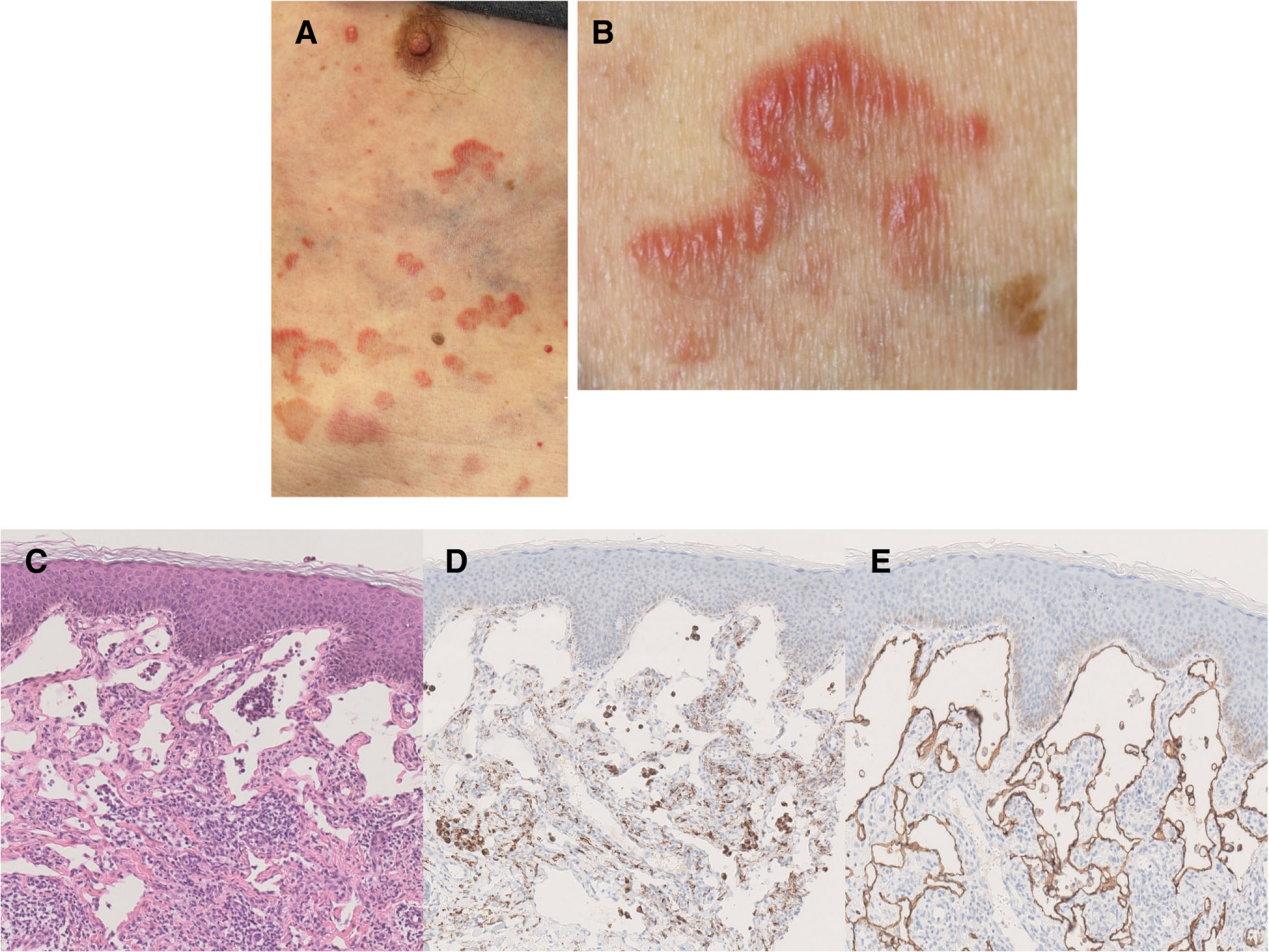
Clinical appearance. A reddish-brown plaque with edema was present on the right side of the trunk of his body (**a**, **b**). Histopathological findings of the patient’s skin biopsy specimens (magnification, 200×). Inflammatory cells, including lymphocytes, plasma cells and macrophages, were present in the dilated vessels (**c**). Immunostaining revealed aggregates of CD68 positive histiocytes (**d**) and endothelial cells lining the vessels were positive for D2–40 (**e**)

**Fig. 3:**
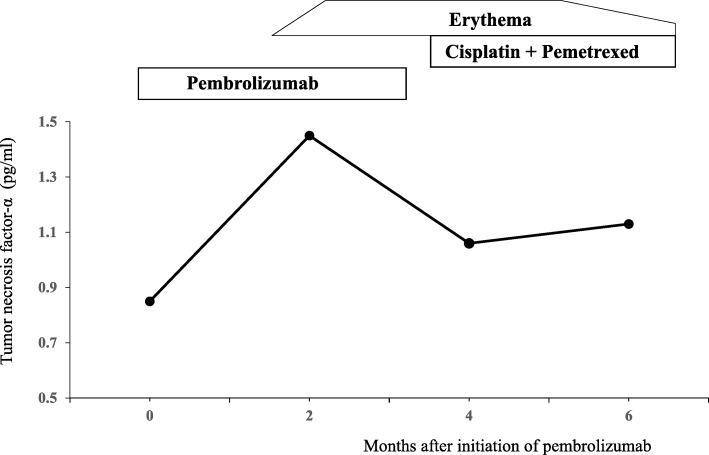
Clinical course of the case study. The TNF-α level was increased during pembrolizumab therapy. After starting cisplatin and pemetrexed treatment, the TNF-α level was decreased

**Fig. 4:**
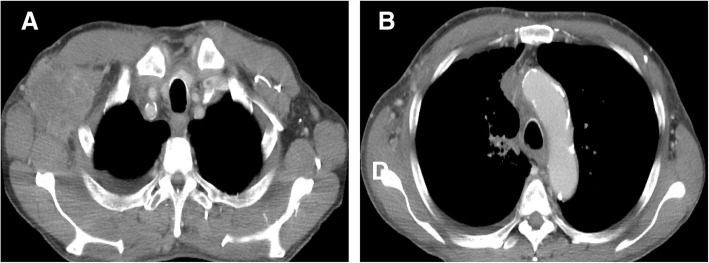
After 4 cycles of pembrolizumab administration, the size of the right axial lymph node metastasis was increased (**a**, **b**)

A 67-year-old man who was a current smoker presented with an edematous right arm and face in our hospital. A chest computed tomography (CT) scan revealed a tumor of approximately 40 mm in diameter in the right upper lobe, with right axial and mediastinal lymph node metastases, and pleural effusion (Fig. 1a and b). According to the findings of a transbronchial lung biopsy and systemic survey, he was diagnosed with adenocarcinoma corresponding to clinical T4N3M1c (stage IVB: 8th edition of UICC TNM staging). An epidermal growth factor receptor mutation and rearranged anaplastic lymphoma kinase genes were not detected. His tumor had invaded the superior vena cava (SVC), leading to the swelling of his right arm and face, suggesting SVC syndrome. He was treated with palliative radiotherapy consisting of a total dose of 30 Gy for SVC syndrome. After irradiation, the size of the tumor in the right upper lobe was slightly decreased (Fig. 1c and d). Immunohistochemistry using the 22C-3 antibody revealed the high expression of PD-L1 and a TPS of 75%. He did not have a personal or family history of any autoimmune conditions and autoimmune related antibodies such as anti Jo-1 antibody, anti-thyroid peroxidase antibody, anti-thyroid stimulating hormone antibody, free T3, free T4, rheumatoid factor (RF), anti-acetylcholine receptor antibody, antinuclear antibody and anti-glutamic acid decarboxylase antibody did not show abnormal findings. Subsequently, pembrolizumab (200 mg/body, every 3 weeks) was initiated as the first-line therapy. Approximately 2.5 months after treatment with pembrolizumab, he presented with an asymptomatic, poorly demarcated 1–3 cm erythematous plaque over the right trunk of his body, which gradually developed in size (Fig. 2a and b). He had no symptoms and his blood examination test results showed no remarkable changes. Therefore, pembrolizumab therapy was continued. Histopathologic examination from a skin biopsy showed ectatic dermal lymphatics with intraluminal aggregations of histiocytes (Fig. 2**c),** which were positive for CD68 and lymphatic vessels that were positive for podoplanin (D2–40) (Fig. 2d and e). We ultimately diagnosed him as ILH based on the clinical and histopathological findings. RF and anti-cyclic citrullinated peptide (CCP) antibody were checked after the appearance of erythematous plaques; however, they were negative. Laboratory results revealed that TNF-α levels were increased after 2 months of pembrolizumab treatment (Fig. 3). After 4 cycles of pembrolizumab treatment, the size of the tumor in right upper lobe had decreased. However, the tumor in the axial lymph node progressed (Fig. 4a and b) and his right arm swelling worsened. Therefore, the treatment was changed to cisplatin (75 mg/m^2^) and pemetrexed (500 mg/m^2^) as second-line therapy. After 2 cycles of chemotherapy, he maintained a partial response without any severe adverse events and ILH was gradually resolved with topical steroid therapy.

## Discussion

Cutaneous ir-AEs present with a wide range of clinical appearances. Hwang et al. [6] reported that 49% of melanoma patients undergoing anti-PD-1 therapy developed dermatologic toxicity. Curry et al. categorized four types of cutaneous ir-AEs as inflammatory, immunobullous, alteration of epidermal keratinocytes, and alteration of epidermal melanocytes [7]. Among cutaneous ir-AEs, skin rash (inflammatory group), pruritus (inflammatory group) and vitiligo (alteration of epidermal melanocytes group) are frequently observed.

ILH is a rare, dermatologic disorder that represents the accumulation of histiocytes within the ectatic lymphatics. ILH was first reported in 1994 [8] as dilated dermal vessels containing collections of mononuclear histiocytes (macrophages) within their lumina. In 1999, Rieger et al. [9] reported two similar patients, one of which had a history of rheumatoid arthritis (RA). In 2005, Okazaki et al. [10] demonstrated the lymphatic nature of the enlarged vessels by assessing the expression of a lymphatic endothelial marker, D2–40. The concept of ILH was established after these findings were reported. ILH is also defined by the accumulation of CD68 positive histiocytes within dilated lymphatic vessels. Barba et al. [11] summarized 59 ILH cases and 23 cases associated with RA. Clinically, most lesions of ILH develop as isolated asymmetrical erythematous plaques with or without a livedo-like pattern, papules, vesicles, or nodules on the extremities. The cause of ILH remains unknown but might be related to intrinsic lung cancer biology or tumor progression. Barba et al. [11] reported six ILH patients with malignant tumors of the breast, colon, as well as melanoma. Secondary ILH often occurs with RA; therefore, the pathogenesis of this disorder might be associated with chronic inflammation. Regarding its role in RA, chronic inflammation might cause lymphostasis leading to the poor clearance of antigen and localized immune dysfunction, subsequently promoting the stimulation and proliferation of histiocytosis in the lymphatic vessels [12]. In addition, TNF-α, a proinflammatory cytokine and a critical mediator of the inflammation in RA [13], plays a crucial role in the pathogenesis of ILH. A previous study [14] reported the effectiveness of infliximab, an anti-TNF-α antibody, against ILH [15]. Treatment algorithms for managing ir-AEs recommend the use of immune suppressants such as corticosteroids and in more severe cases, the use of infliximab [16]. It was reported that a certain proportion of patients treated with ICIs experienced severe colitis and that infliximab improved colitis [17]. In the current case, TNF-α levels were elevated after pembrolizumab treatment. Therefore, TNF-α might play an important role in the induction of ILH in this case.

He received palliative radiotherapy before pembrolizumab treatment. Recently, radiation therapy was shown to activate immune responses. Abscopal effects were used to describe the phenomenon of tumor regression in untreated metastatic lesions after local treatment, such as radiotherapy [18]. The potential mechanism of abscopal effects might involve the triggering of increased tumor antigen release and presentation to T-cells by irradiation of the lesion, which subsequently enhances immunological responses. Therefore, his risk for ILH might be increased by the radiation treatment. It was reported that the abscopal effect synergized with the presence of immunotherapy [19]. In the PACIFIC study, ir-AEs of any grade were 24.2% in the PD-L1 inhibitor (durvalumab) group and 8.1% in the placebo group [20]. Based on these findings, a synergic effect with radiation therapy and pembrolizumab might induce ILH as well as pembrolizumab alone.

## Conclusion

In summary, this is the first case of secondary ILH induced by pembrolizumab therapy and the first report to the best of our knowledge to confirm the upregulation of TNF-α. Further studies are needed to clarify the mechanisms of Ir-AEs following immunotherapy.

## Abbreviations

ICIs: immune checkpoint inhibitors
ILH: Intralymphatic histiocytosis
NSCLC: non-small-cell lung cancer
PD-1: anti-programmed death
PD-L1: programmed death ligand 1
TNF-α: tumor necrosis factor α
